# Exploratory analysis of the knowledge, attitudes and perceptions of healthcare workers about arboviruses in the context of surveillance in the Republic of Guinea

**DOI:** 10.1371/journal.pntd.0011814

**Published:** 2023-12-04

**Authors:** Salifou Talassone Bangoura, Castro Gbêmêmali Hounmenou, Sidikiba Sidibé, Saidouba Cherif Camara, Aminata Mbaye, Marie-Marie Olive, Alioune Camara, Alexandre Delamou, Alpha-Kabinet Keita, Eric Delaporte, Nagham Khanafer, Abdoulaye Touré

**Affiliations:** 1 Centre de Recherche et de Formation en Infectiologie de Guinée (CERFIG), Gamal Abdel Nasser University, Conakry, Republic of Guinea; 2 Department of Public Health, Gamal Abdel Nasser University, Conakry, Republic of Guinea; 3 Department of Pharmaceutical and Biological Sciences, Gamal Abdel Nasser University, Conakry, Republic of Guinea; 4 African Centre of Excellence in the Prevention and Control of Communicable Diseases (CEA-PCMT), Faculty of Sciences and Health Techniques, Gamal Abdel Nasser University, Conakry, Republic of Guinea; 5 CIRAD, UMR ASTRE, F-34398 Montpellier, France; 6 ASTRE, University of Montpellier, CIRAD, INRAE, Montpellier, France; 7 Recherches Translationnelles sur le VIH et les Maladies Infectieuses (TransVIHMI), University of Montpellier, Institut National de la Santé et de la Recherche Médicale (INSERM), Institut de Recherche pour le Développement (IRD), 34394 Montpellier, France; 8 PHE3ID Team, Centre International de Recherche en Infectiologie (CIRI), Inserm U1111, CNRS UMR5308, ENS de Lyon, Université de Lyon 1, Lyon, France; 9 Hygiene, Epidemiology and Prevention Unit, Edouard Herriot Hospital, Hospices Civils de Lyon, Lyon, France; The University of Sydney School of Veterinary Science, AUSTRALIA

## Abstract

**Background:**

The escalating risk and contemporary occurrences of arbovirus infections prompt a critical inquiry into the ability of nations to execute efficient surveillance systems capable to detect, prevent and respond to arbovirus outbreaks. Healthcare workers (HCWs) are the major actors in the surveillance of infectious diseases with epidemic potential. The objective of this study was to evaluate the knowledge, attitudes and perceptions of HCWs regarding arboviruses in the public health facilities of Conakry, Guinea.

**Methods:**

A cross-sectional survey was conducted during the from December 27, 2022, to January 31, 2023, encompassing from public health facilities in Conakry. The data collection process encompassed various aspects, including the characteristics of health facilities, socio-demographic and professional attributes of HCWs, the information received concerning arboviruses and the sources of information, as well as a series of inquiries designed to evaluate their knowledge, attitudes and perceptions. Subsequently, scores were computed for knowledge, attitude and perception. To identify the factors influencing the knowledge, attitudes, and perceptions of HCWs regarding arboviruses, Decision Tree and Inference Conditional Tree models were used.

**Results:**

A total of 352 HCWs participated in the study, comprising 219 from national hospitals, 72 from municipal hospitals and 61 from primary health centers. More than half of the respondents (54.3%) had never received information on arboviruses. Only 1% of the respondents had good knowledge about arboviruses, 95.7% had a negative attitude about arboviruses. Moreover, nearly 60% of the respondents had a moderate perception and 24.1% had a good perception. The analysis revealed significant associations between the knowledge and attitudes of respondents concerning arboviruses and their years of professional experience and age.

**Conclusion:**

This study highlights the imperative requirement for comprehensive training targeting HCWs to enhance their capacity for early case detection within healthcare facilities. Additionally, there is a crucial need for analogous studies adopting a mixed-methods approach across all healthcare regions in Guinea.

## Author summary

Arboviruses, including yellow fever, dengue, Zika, chikungunya, and Rift Valley fever, constitute viral diseases transmitted by blood-sucking arthropod vectors, such as mosquitoes, ticks, and biting midges, to vertebrate hosts. In many countries, particularly within Africa, the prevalence of these arboviruses remains widely underestimated due to the absence of effective surveillance systems, often resulting in the conflation of arboviral infections with other infectious diseases. Nevertheless, sub-Saharan Africa is prone to periodic arbovirus-linked epidemics, propelled by factors such as the expansion of vector habitats, viral genetic mutations, anthropogenic influences, inadequate healthcare services, commercial transportation, and climate fluctuations. While Guinea has predominantly reported suspected cases of yellow fever, widespread arbovirus-related epidemics have yet to be documented. Nevertheless, it is imperative for healthcare workers to be well-informed and prepared for prospective epidemics. The assessment of the knowledge, attitudes, and perceptions of healthcare workers can serve as a catalyst to heighten awareness, bolster surveillance mechanisms, and enhance the capabilities of public health facilities in the prevention and mitigation of potential epidemics.

## Introduction

In recent decades, viral diseases transmitted by arthropods, including dengue, yellow fever, Japanese encephalitis, chikungunya and Rift Valley fever, have persistently posed a substantial threat to both global public health and socio-economic development [1,2]. These diseases collectively constitute over 17% of infectious diseases, and are annually responsible for in excess of one million fatalities on a global scale [3]. Dengue fever, notably lethal in its haemorrhagic manifestation, stands as the most prevalent arbovirus, afflicting an estimated 390 million individuals annually and resulting in approximately 25,000 fatalities [4].

Many of these arbovirus infections, often displaying an asymptomatic nature or limited to relatively mild influenza-like syndromes such as moderate fever, conjunctivitis, rash, headache, myalgia, etc., tend to remain inconspicuous. However, they possess the potential to escalate or disseminate, leading to profound health, societal, and economic repercussions [5]. Recently, outbreaks were notified in many African countries as dengue fever in Tanzania [6] and Sudan [7], chikungunya in the Republic of Congo [8], Zika virus in Cape Verde [9] and Angola [10] and Yellow fever in Nigeria, Ethiopia, Uganda, South Sudan, Guinea and Senegal [11].

The increasing menace posed by arbovirus infections, along with recent outbreaks, instigates an essential inquiry into the proficiency of countries in executing proficient surveillance operations [12]. In 2018, an assessment of the existing epidemiological and entomological surveillance resources across 16 West African nations, which encompassed Guinea, unveiled the region’s latent capacity for epidemiological, entomological, and vector control surveillance. Nevertheless, it is evident that these capacities require significant enhancement to guarantee the prompt detection of arbovirus infections and to bolster preparedness in mitigating potential outbreaks [12].

In Guinea, diligent surveillance mechanisms are in place for monitoring diseases such as dengue, yellow fever and Rift Valley fever, all of which constitute integral components of the nine priority zoonotic diseases [13]. However, it is essential to acknowledge that a multitude of widespread zoonotic pathogens, encompassing both viral and bacterial agents, give rise to diseases of varying severity, often characterized by clinical symptoms akin to those exhibited by arboviruses [14], which remain misdiagnosed and/or underdiagnosed. Research has indicated that arbovirus diseases such as yellow fever, make a substantial contribution to the morbidity in Guinea [14–16]. In this context, there is a distinct imperative for heightened focus on preventive measures and the early identification of cases, aimed at averting the potential emergence of a widespread epidemic. This responsibility inherently falls upon health authorities, as well as healthcare workers who are engaged in surveillance activities geared towards the expeditious detection and response to health events, notably encompassing arbovirus diseases, which hold significance for both their communities and the nation at large [17]. It requires a holistic understanding of the reservoir, the vector, the mode of transmission, preventive measures and also the appropriate attitudes to adopt when caring for patients. Assessing the knowledge, attitudes and perceptions of HCWs plays a central role in arboviruses surveillance. The main objective of this study was to assess the knowledge, attitudes and perceptions of HCWs concerning arbovirus infections in Conakry, Guinea.

## Methods

### Ethics statement

The study protocol was approved by the Guinean Health Research Ethics Committee (N°: 023/CNERS/23). An information letter was sent to the communal health directors of the 5 municipalities before the start of the study, and authorisation to collect data from the heads of the health facilities was obtained. In addition, the objective and the expected results of the study were explained to the respondents through an information note on the first page of the questionnaire. Participation in the study was conditional on obtaining free and informed consent. All information were collected anonymously and respondents’ free and informed verbal consent was obtained.

### Study area

**Fig 1:**
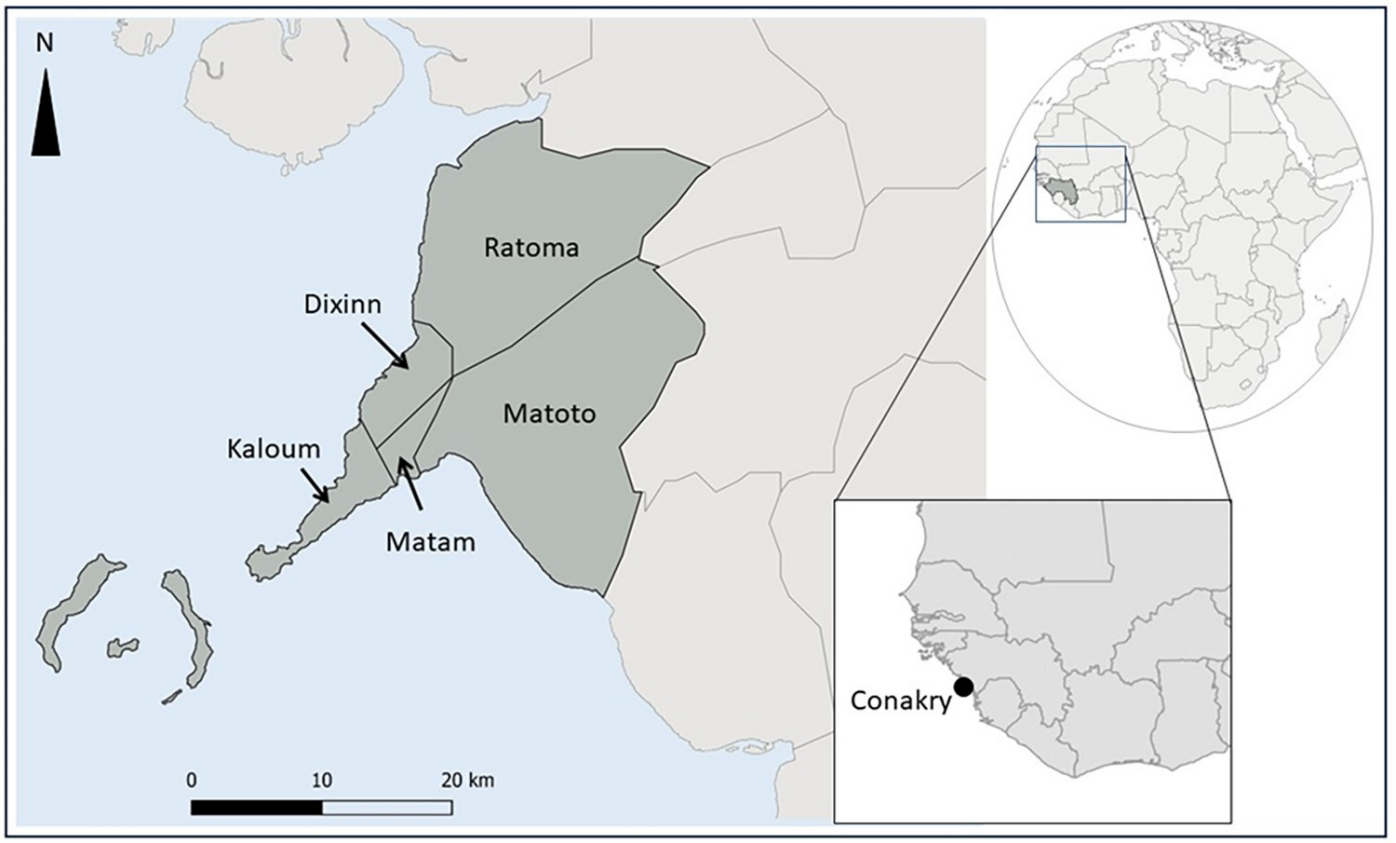
Map of Conakry and its five communes (Map created with QGIS software: source of administrative boudaries map layer: https://www.gadm.org/; Link to the GADM license: https://www.gadm.org/license.html).

This study was carried out in public health facilities in Conakry. Conakry is the capital of the Republic of Guinea located on the country’s southwest coast and covering an area of 450 square kilometers. With a population of 1,660,973, Conakry is the second densely populated region in Guinea [18]. Administratively, Conakry is divided into five urban municipalities: Kaloum, Dixinn, Matam, Matoto and Ratoma (Fig 1). Regarding the repartition of health professionals, 28.4% of HCWs are concentrated in Conakry [19].

### Study design and population

A cross-sectional survey was conducted between December 2022 and January 2023 in public health facilities in Conakry aiming to assess the knowledge, attitudes and perceptions of HCWs about arbovirus infections. The study was performed in public health facilities at different levels: three hospitals at national level (Donka in the municipalities of Dixinn, Ignace Deen in the municipalities of Kaloum and Sino-Guinean Hospital in the municipality of Ratoma); five municipal hospitals at secondary level (Kaloum in the municipality of Kaloum, Minière in the municipality of Dixinn, Matam in the municipality of Matam, Coleah in the municipality of Matam, Flamboyant in the municipality of Ratoma and Ratoma in the municipality of Ratoma) and 18 primary health centers at primary level, including three in the municipalities of Kaloum and Dixinn, one in the municipality of Matam, seven in the municipality of Matoto and eight in the municipality of Ratoma. The study concerned all HCWs in the department of the public health facilities in Conakry.

### Outcome criteria

#### Knowledge

The knowledge of respondents regarding arboviruses was assessed using 21 questions on: definition, types of arboviruses, vectors, mode of transmission, patient management (symptoms, associated complications, diagnostic methods and treatment) and protection measures (S1 Questionnaire). Each correct answer had a value of "1" and the incorrect or don’t know answer had a value of "0". The values were added to obtain a total score from 0 to 21 points which was categorized according to the modified Bloom criteria [20–22]: good knowledge (80–100%) if the score was between 17 and 21 points, moderate (50–79%) when the score was between 11 and 16 points, poor (<50%) in case of a score less than 11 points. Then, we assessed the knowledge of respondents by item (Type, vector and mode of transmission; Medical management and Preventive measures). Following the same principle, each correct answer had a value of "1" and the incorrect or don’t know answer had a value of "0". The scores obtained by each participant were added and categorised as follows: good knowledge (80–100%) if the score was between 17 and 21 points, moderate (50–79%) when the score was between 11 and 16 points, poor (<50%) in case of a score less than 11 points. Then, we assessed the knowledge of respondents by item (Type, vector and mode of transmission; Medical management and Preventive measures). Following the same principle, each correct answer had a value of "1" and the incorrect or don’t know answer had a value of "0". The scores obtained by each participant were added and categorised as good knowledge if the participants had 80 to 100% of the total score per item, moderate knowledge if they had 50 to 79% of the total score per item and poor knowledge if they had less than 50% of the total score per item.

#### Attitudes

Attitudes about arbovirus-related diseases were assessed using eight questions focusing on the following items: the risk of transmission, diagnosis, treatment and prevention. the response was classified according to three scale measures with agreement, disagreement and don’t know (S1 Questionnaire). Each correct answer was coded as "1" and the incorrect or neutral answer was coded as "0". An overall score above six (80%) was considered as a positive attitude, while a score less than or equal to six (<80%) was considered as a negative attitude. For each item, the attitudes of the respondents was evaluated. Each correct answer was coded as "1" and the incorrect or neutral answer as "0". Respondents’ attitudes per item were considered positive if they obtained 80% of the total score per item and negative if they obtained less than 80% of the total score per item.

#### Perceptions

They were measured with 16 questions focusing on severity, exposure and protection measures (S1 Questionnaire). Responses were ranked on a scale from 1 to 5. The total score varied from 16 to 80 points. The respondents’ perceptions were categorized according to the modified Bloom criteria [20–22]: good perception if the score is between 80 and 100%, moderate if the score is between 50 and 79%, low if the score is less than 50%. Respondents’ perceptions were assessed for each item. Respondents’ perceptions per item were added and categorized as follows: good perception if the score is between 80 and 100% of the total score per item, moderate if the score is between 50 and 79% of the total score per item, low if the score is less than 50% of the total score per item.

### Collected data

Data were collected during working days in the gynecology, internal medicine, oto-rhino-laryngology, neurology, pulmonology, pediatrics, rheumatology, medical emergency, dermatology, hematology, infectious diseases and primary curative consultation department from self-administration of a standardized in the HCWs present during the survey period. The questionnaire was pre-tested to ensure that it would be well understood by the respondents. In each participating department, the questionnaire was distributed to all present HCWs and was picked up the same day.

The data were related to characteristics of the health facility including: type of facility (national hospital, Municipal hospitals, primary health centers), socio-demographic and professional characteristics including: gender, age, grade and professional experience (in years), information received on arbovirus diseases and sources of information.

### Statistical analysis

The categorical variables were summarized with percentages and the quantitative variables were described by median and interquartile range. Pearson’s Chi-squared test or Fisher’s exact test was used to determine significant differences between categorical variables according to their condition of use. To evaluate the relationships between these variables and the different outcomes, tree-structured models (Decision Tree and Inference Conditional Tree) were used. The best tree model was selected based on the high value of Accuracy and the low value of no information rate [23,24]. Before implementing the models, the oversampling technique was used to adjust for class balance. R Studio 3.5.3 software was used for data processing and analysis. Map created with QGIS software (source of administrative boudaries map layer: https://www.gadm.org/; Link to the GADM license: https://www.gadm.org/license.html).

## Results

A total of 352 HCWs participated in this survey, of which 219 were from university hospitals, 72 from municipal hospitals and 61 from primary health centers. The respondents had a median age of 33 years (IQR: 29–38) and were mostly male (62%, n = 218). Three quarters of respondents (72.7%) were physicians and 27.3% were medical student. The median years of experience of respondents was 4.6 years (IQR: 3.0–8.0). This study revealed that a substantial majority of respondents (54.3%) had never received information about arboviruses. Among the remaining respondents (n = 161, 45.7%), the majority received this information several years prior primarily through medical Journal/scientific congresses (55.9%), colleagues on duty (18%), or media sources (26.1%). Notably, a high percentage of participants, accounting for 91.2%, expressed a specific need for information concerning arboviruses, particularly focusing on management (75.9%), preventive measures (68.2%), modes of transmission (65.1%), and clinical features (59.1%) (Table 1).

**Table 1:** Socio-demographic and professional characteristics of healthcare workers in the health facilities of Conakry.

Characteristics	n (%)
**Type of health facility** (N = 352)	
National Hospitals	219 (62.2)
Municipal hospitals	72 (20.5)
Primary health centers	61 (17.3)
**Gender** (N = 352)	
Female	134 (38.1)
Male	218 (61.9)
**Age (Years)** (N = 352)	
<30	101 (28.7)
30–35	128 (36.4)
>35	123 (34.9)
**Grade** (N = 352)	
General practitioner	177 (50.3)
Specialized clinician	79 (22.4)
Medical thesis student	96 (27.3)
**Years of experience (Years)** (N = 352)	
< 5	176 (50.0)
5–10	131 (37.2)
>10	45 (12.8)
**Information on arboviruses received previously** (N = 352)	
No	191 (54.3)
Yes	161 (45.7)
**When this information was received** (N = 161)	
One month ago	31 (19.2)
Several months ago	28 (17.4)
Several years ago	102 (63.4)
**Main source of this information** (N = 161)	
Medical Journal/scientific congresses	90 (55.9)
Media (radio/TV/online media)	42 (26.1)
Service colleagues	29 (18.0)
**Information needs on arboviruses** (N = 352)	
No	31 (8.8)
Yes	321 (91.2)
**Types/categories of needed information** (N = 321)	
Management	267 (75.9)
Prevention measures	240 (68.2)
Mode of transmission	229 (65.1)
Clinical characteristics	208 (59.1)
Physiopathology	185 (52.6)
Epidemiology	174 (49.4)
Policy Directives	148 (42.0)

**Fig 2:**
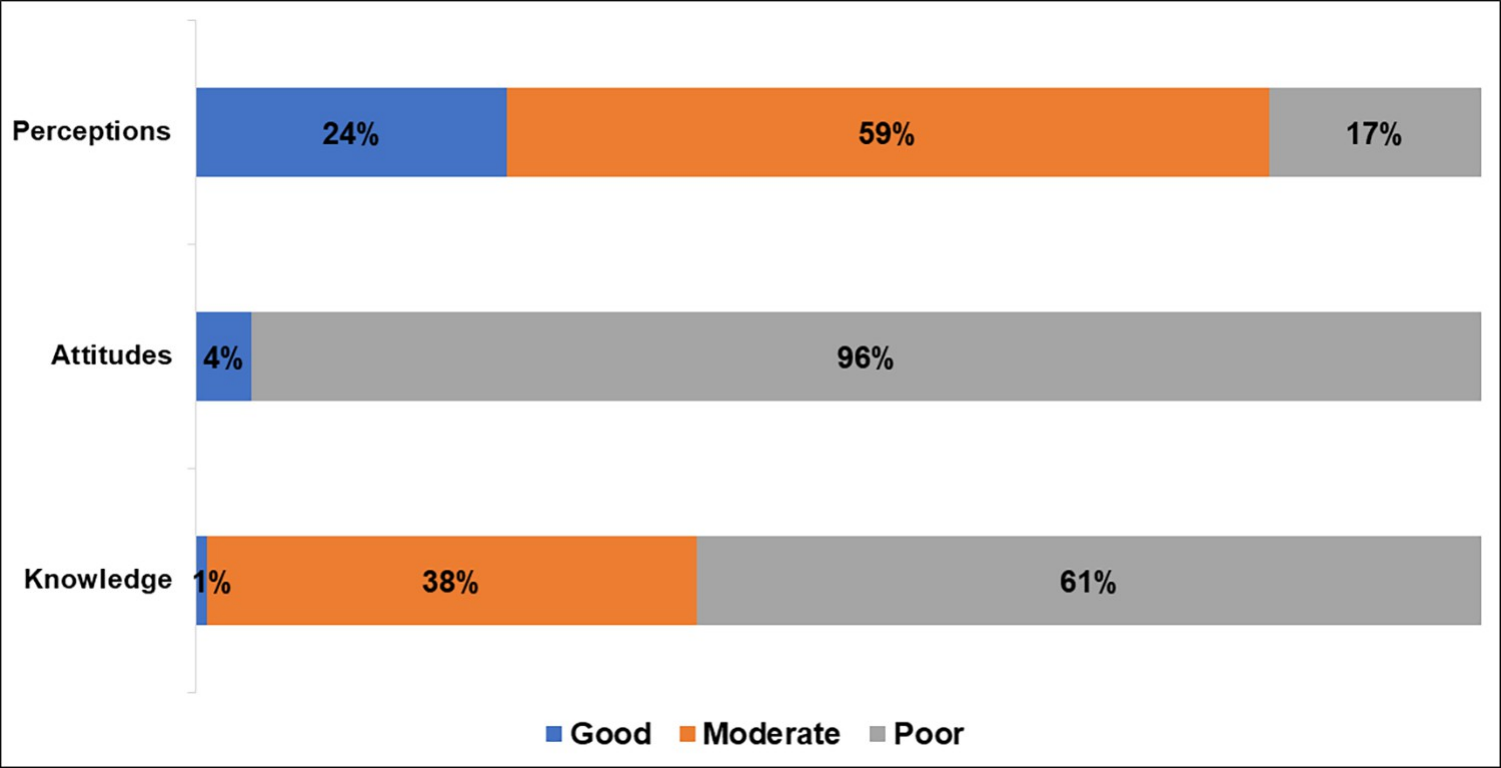
Knowledge, attitudes and perceptions of arboviruses among healthcare workers in public health facilities in Conakry.

Overall, almost two-thirds of respondents (61%) had low knowledge, 38% had moderate knowledge and only 1% had good knowledge about arboviruses. Similarly, almost all respondents (96%) had a negative attitude towards arboviruses. However, more than half (59%) of respondents had moderate perception, and only 24% had good perception (Fig 2).

### Knowledge

HCWs in municipal hospitals (61.1%) and primary health centers (50.8%) had a lower knowledge score on the types, vectors and modes of transmission of arboviruses than those in national hospitals (p = 0.041). Additionally, it was observed that a higher proportion of males with good knowledge to the management (p<0.001) and prevention of arboviruses (p = 0.028) in comparison to females. Of significance, more than two-thirds of medical students had poor knowledge of the management of arboviruses and only 11.4% of specialized clinicians had good knowledge (p = 0.001). Similarly, more than half of the less experienced HCWs had poor on the management of arboviruses (p<0.001) (Table 2)

**Table 2:** Healthcare workers’ knowledge of the types, vectors, modes of transmission, medical management and preventive measures for arboviruses.

Characteristics	Type, vector and mode of transmission				Medical management				Preventive measures			
	Poor Knowledge n(%)	Moderate Knowledge n(%)	Good knowledge n(%)	*p	Poor Knowledge n(%)	Moderate Knowledge n(%)	Good knowledge n(%)	*p	Poor Knowledge n(%)	Moderate Knowledge n(%)	Good knowledge n(%)	*p
**Overall**	172 (48.9)	145 (41.2)	35 (9.9)		177 (50.3)	154 (43.8)	21 (6.0)		258 (73.3)	91 (25.9)	3 (0.9)	
**Type of health facility**				0.041				0.11				0.20
National Hospitals	97 (44.3)	103 (47.0)	19 (8.7)		109 (49.8)	101 (46.1)	9 (4.1)		155 (70.8)	62 (28.3)	2 (0.9)	
Municipal Hospitals	44 (61.1)	19 (26.4)	9 (12.5)		40 (55.6)	24 (33.3)	8 (11.1)		60 (83.3)	12 (16.7)	0 (0)	
Primary health centers	31 (50.8)	23 (37.7)	7 (11.5)		28 (45.9)	29 (47.5)	4 (6.6)		43 (70.5)	17 (27.9)	1 (1.6)	
**Gender**				0.055				<0.001				0.028
Female	76 (56.7)	45 (33.6)	13 (9.7)		85 (63.4)	48 (35.8)	1 (0.7)		108 (80.6)	26 (19.4)	0 (0)	
Male	96 (44.0)	100 (45.9)	22 (10.1)		92 (42.2)	106 (48.6)	20 (9.2)		150 (68.8)	65 (29.8)	3 (1.4)	
**Age (Years)**				0.80				0.012				0.050
<30	49 (48.5)	43 (42.6)	9 (8.9)		62 (61.4)	35 (34.7)	4 (4.0)		76 (75.2)	25 (24.8)	0 (0)	
30–35	67 (52.3)	50 (39.1)	11 (8.6)		68 (53.1)	53 (41.4)	7 (5.5)		101 (78.9)	27 (21.1)	0 (0)	
>35	56 (45.5)	52 (42.3)	15 (12.2)		47 (38.2)	66 (53.7)	10 (8.1)		81 (65.9)	39 (31.7)	3 (2.4)	
**Grade**				0.80				0.001				0.066
General practitioners	92 (52.0)	68 (38.4)	17 (9.6)		80 (45.2)	88 (49.7)	9 (5.1)		133 (75.1)	42 (23.7)	2 (1.1)	
Specialized clinicians	34 (43.0)	37 (46.8)	8 (10.1)		33 (41.8)	37 (46.8)	9 (11.4)		49 (62.0)	29 (36.7)	1 (1.3)	
Medical thesis students	46 (47.9)	40 (41.7)	10 (10.4)		64 (66.7)	29 (30.2)	3 (3.1)		76 (79.2)	20 (20.8)	0 (0)	
**Years of experience**				0.14				<0.001				0.40
< 5	80 (45.5)	80 (45.5)	16 (9.1)		101 (57.4)	70 (39.8)	5 (2.8)		131 (74.4)	45 (25.6)	0 (0)	
5–10	74 (56.5)	43 (32.8)	14 (10.7)		65 (49.6)	54 (41.2)	12 (9.2)		96 (73.3)	33 (25.2)	2 (1.5)	
>10	18 (40.0)	22 (48.9)	5 (11.1)		11 (24.4)	30 (66.7)	4 (8.9)		31 (68.9)	13	1 (2.2)	

*p-value associated for Pearson’s Chi-squared test or Fisher’s exact test

**Fig 3:**
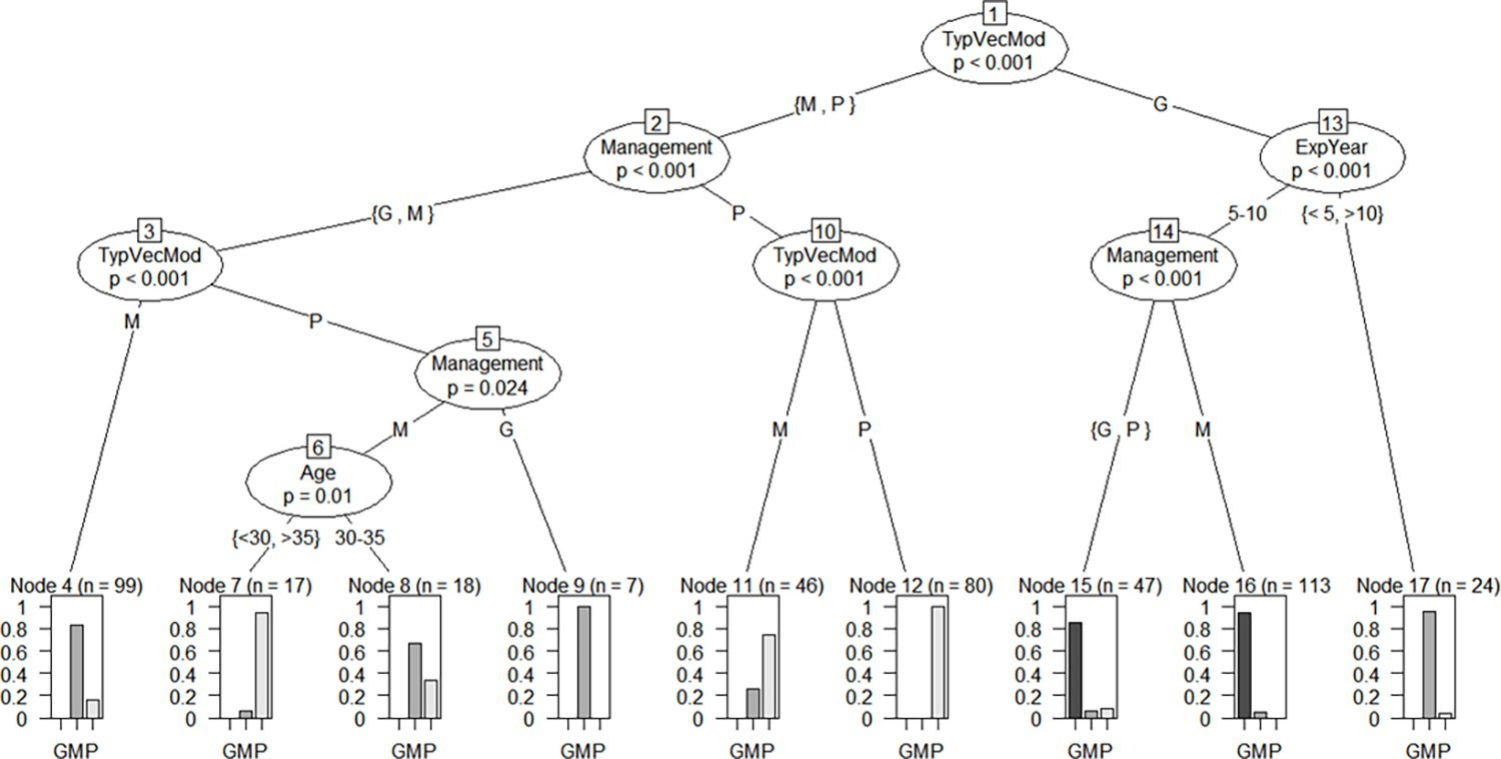
Conditional inference tree model on the knowledge of healthcare workers in public health facilities in Conakry Abbreviation: G = Good knowledge; M = Moderate knowledge; P = Poor knowledge; TypVecMod = Respondents’ knowledge of type, vector and mode of transmission; Management = Knowledge of medical management; and ExpYear = Year of experience.

The overall knowledge of respondents concerning arboviruses was significantly associated with their age and professional experience. Specifically, those with 5–10 years of experience were more likely to have a good knowledge of arboviruses, including a good knowledge of the types, vectors and mode of transmission (Node 15 and 16) (p<0.001). However, the tree predicts poor knowledge of arboviruses among respondents aged under 30 and those aged over 35 with poor knowledge of the types, vectors, modes of transmission and moderate knowledge of the medical management of arboviruses (Node 7) (p = 0.01) (Fig 3).

### Attitudes

About 64% of healthcare workers in primary healthcare centers had a more positive attitude than those in other healthcare facilities about the risk of transmission of arboviruses (p = 0.044), but only 18% had a more positive attitude about the management and prevention of arboviruses (p = 0.012). Furthermore, general practitioners (59.3%) and specialized clinicians (58.2%) had a more positive attitude towards the risk of transmission than medical students (p = 0.037) (Table 3).

**Table 3:** Healthcare workers’ attitudes on the risk of transmission, diagnosis, prevention and treatment of arboviruses.

Characteristics	Risk of transmission of arboviruses			Diagnosis/prevention			Treatment		
	Negative attitude n(%)	Positive attitude n(%)	*p	Negative attitude n(%)	Positive attitude n(%)	*p	Negative attitude n(%)	Positive attitude n(%)	*p
**Overall**	159 (45.2)	193 (54.8)		251 (71.3)	101 (28.7)		346 (98.3)	6 (1.7)	
**Type of health facility**			0.044			0.012			0.3
National Hospitals	96 (43.8)	123 (56.2)		144 (65.8)	75 (34.2)		213 (97.3)	6 (2.7)	
Municipal Hospitals	41 (56.9)	31 (43.1)		57 (79.2)	15 (20.8)		72 (100)	0 (0)	
Primary health centers	22 (36.1)	39 (63.9)		50 (82.0)	11 (18.0)		61 (100)	0 (0)	
**Gender**			0.40			0.3			0.9
Female	64 (47.8)	70 (52.2)		100 (74.6)	34 (25.4)		132 (98.5)	2 (1.5)	
Male	95 (43.6)	123 (56.4)		151 (69.3)	67 (30.7)		214 (98.2)	4 (1.8)	
**Age (Years)**			0.015			0.9			0.2
<30	46 (45.5)	55 (54.5)		71 (70.3)	30 (29.7)		98 (97.0)	3 (3.0)	
30–35	69 (53.9)	59 (46.1)		93 (72.7)	35 (27.3)		125 (97.7)	3 (2.3)	
>35	44 (35.8)	79 (64.2)		87 (70.7)	36 (29.3)		123 (100)	0 (0)	
**Grade**			0.037			0.2			0.4
General practitioners	72 (40.7)	105 (59.3)		127 (71.8)	50 (28.2)		174 (98.3)	3 (1.7)	
Specialized clinicians	33 (41.8)	46 (58.2)		51 (64.6)	28 (35.4)		79 (100)	0 (0)	
Medical students	54 (56.2)	42 (43.8)		73 (76.0)	23 (24.0)		93 (96.9)	3 (3.1)	
**Years of experience (Years)**			0.062			0.3			0.9
< 5	83 (47.2)	93.0 (52.8)		120 (68.2)	56 (31.8)		172 (97.7)	4 (2.3)	
5–10	63 (48.1)	68 (51.9)		100 (76.3)	31 (23.4)		129 (98.5)	2 (1.5)	
>10	13 (28.9)	32 (71.1)		31 (68.9)	14 (31.1)		45 (100)	0 (0)	

*p-value associated for Pearson’s Chi-squared test or Fisher’s exact test

**Fig 4:**
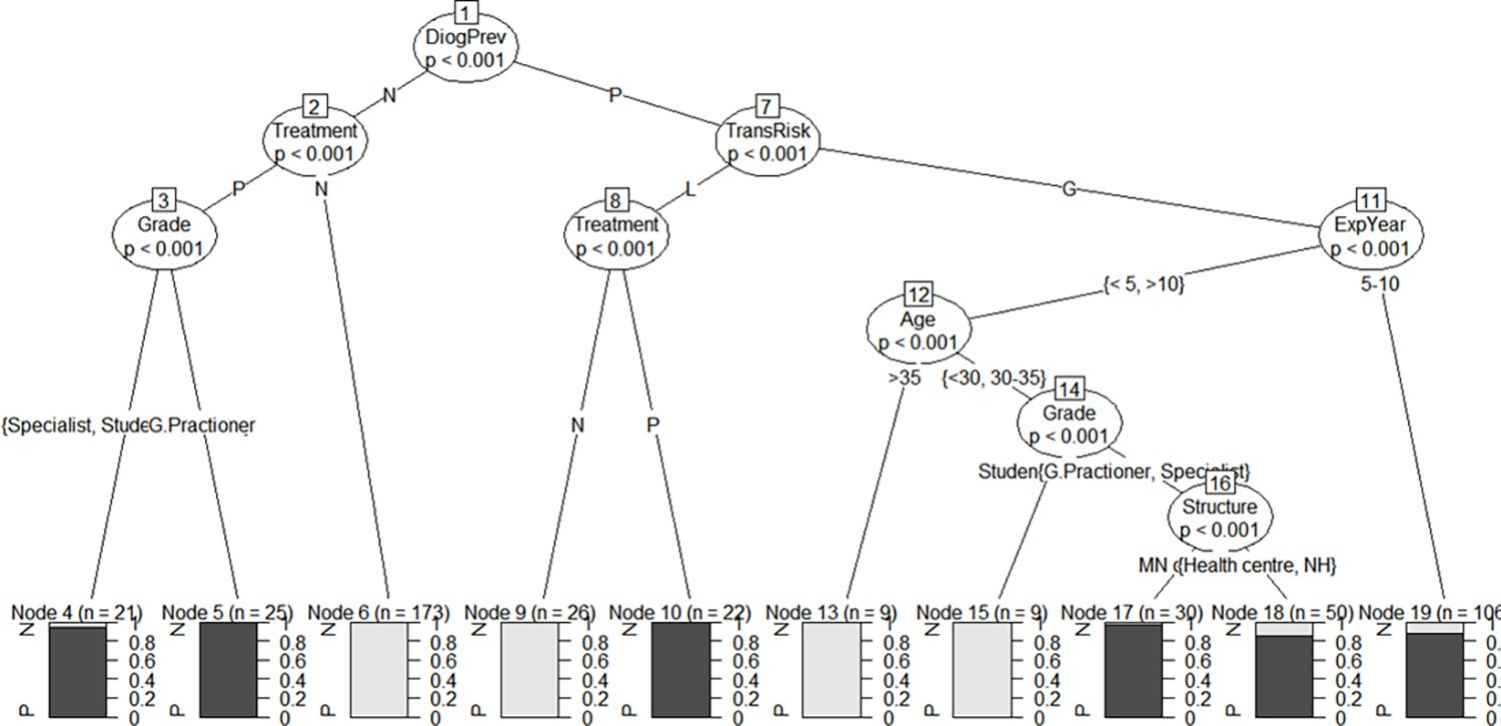
Conditional inference tree model on the attitudes of healthcare workers in public health facilities in Conakry Abbreviations: N = Negative attitude; P = designates Positive attitude; DiagPrev = d respondents’ attitudes on diagnosis and prevention of arboviroses; TransRisk = attitudes on transmission risks; ExpYear = Year of experience; G.Practioner = General practitioner; and NH = National Hospital.

The attitudes of healthcare workers regarding arboviruses were related to their grade, age and professional experience. Notably, both general practitioners and specialists had a positive attitude regardless of their health structure (Node 17 and 18) (p<0.001). Similarly, the model predicts a positive attitude in HCWs with 5 to 10 years’ experience (Node 19) (p<0.001). In contrast, the model predicts a negative attitude in respondents aged over 35 with less than 5 years’ professional experience (Node 13) and in young participants still ungraduated (Node 15) (p<0.001) (Fig 4).

### Perceptions

The perception of exposure and methods of prevention of arboviruses differ according to the type of public health facilities. HCWs in health centers had the highest perception score than those in other health facilities. Three quarters of healthcare workers (75.4%) in health centers had a good perception of exposure to arboviruses, compared with 70.8% of HCWs in municipal hospitals and 63.9% of those in national hospitals (p = 0.004). Similarly, specialized clinicians had a good perception (25.3%) of preventive measures than general practitioners (14.7%) and medical students (14.6%; p = 0.012). Additionally, the perception of exposure to arboviruses was better in participants with more than 10 years of experience (p = 0.004) (Table 4).

**Table 4:** Healthcare workers’ perception of the severity, risk of exposure and prevention measures for arboviruses.

Characteristic	Gravity				Exposition				Prevention measures			
	Low perception n(%)	Moderate perception n(%)	Good perception n(%)	*p	Low perception n(%)	Moderate perception n(%)	Good perception n(%)	*p	Low perception n(%)	Moderate perception n(%)	Good perception n(%)	*p
**Overall**	15 (4.3)	108 (30.7)	229 (65.1)		23 (6.5)	92 (26.1)	237 (67.3)		115 (32.7)	177 (50.3)	60 (17.0)	
**Type of health facility**				0.09				0.004				0.004
National Hospitals	6 (2.7)	76 (34.7)	137 (62.6)		9 (4.1)	70 (32.0)	140 (63.9)		67 (30.6)	113 (51.6)	39 (17.8)	
Municipal Hospitals	6 (8.3)	18 (25.0)	48 (66.7)		7 (9.7)	14 (19.4)	51 (70.8)		29 (40.3)	34 (47.2)	9 (12.5)	
Primary health centers	3 (4.9)	14 (23.0)	44 (72.1)		7 (11.5)	8 (13.1)	46 (75.4)		19 (31.1)	30 (49.2)	12 (19.7)	
**Gender**				0.90				0.40				0.10
Female	5 (3.7)	40 (29.9)	89 (66.4)		8 (6.0)	30 (22.4)	96 (71.6)		46 (34.3)	59 (44.0)	29 (21.6)	
Male	10 (4.6)	68 (31.2)	140 (64.2)		15 (6.9)	62 (28.4)	141 (64.7)		69 (31.7)	118 (54.1)	31 (14.2)	
**Age (Years)**				0.80				0.20				0.064
<30	4 (4.0)	35 (34.7)	62 (61.4)		7 (6.9)	29 (28.7)	65 (64.4)		35 (34.7)	50 (49.5)	16 (15.8)	
30–35	5 (3.9)	39 (30.5)	84 (65.6)		8 (6.2)	40 (31.2)	80 (62.5)		51 (39.8)	60 (46.9)	17 (13.3)	
>35	6 (4.9)	34 (27.6)	83 (67.5)		8 (6.5)	23 (18.7)	92 (74.8)		29 (23.6)	67 (54.5)	27 (22.0)	
**Grade**				0.50				0.40				0.012
General practitioners	6 (3.4)	49 (27.7)	122 (68.9)		9 (5.1)	43 (24.3)	125 (70.6)		52 (29.4)	99 (55.9)	26 (14.7)	
Specialized clinicians	4 (5.1)	25 (31.6)	50 (63.3)		8 (10.1)	19 (24.1)	52 (65.8)		33 (41.8)	26 (32.9)	20 (25.3)	
Medical students	5 (5.2)	34 (35.4)	57 (59.4)		6 (6.2)	30 (31.2)	60 (62.5)		30 (31.2)	52 (54.2)	14 (14.6)	
**Years of experience**				0.067				0.004				0.050
< 5	5 (2.8)	62 (35.2)	109 (61.9)		9 (5.1)	60 (34.1)	107 (60.8)		66 (37.5)	86 (48.9)	24 (13.6)	
5–10	10 (7.6)	34 (26.0)	87 (66.4)		12 (9.2)	27 (20.6)	92 (70.2)		41 (31.3)	67 (51.1)	23 (17.6)	
>10	0 (0)	12 (26.7)	33 (73.3)		2 (4.4)	5 (11.1)	38 (84.4)		8 (17.8)	24 (53.3)	13 (28.9)	

*p-value associated for Pearson’s Chi-squared test or Fisher’s exact test

**Fig 5:**
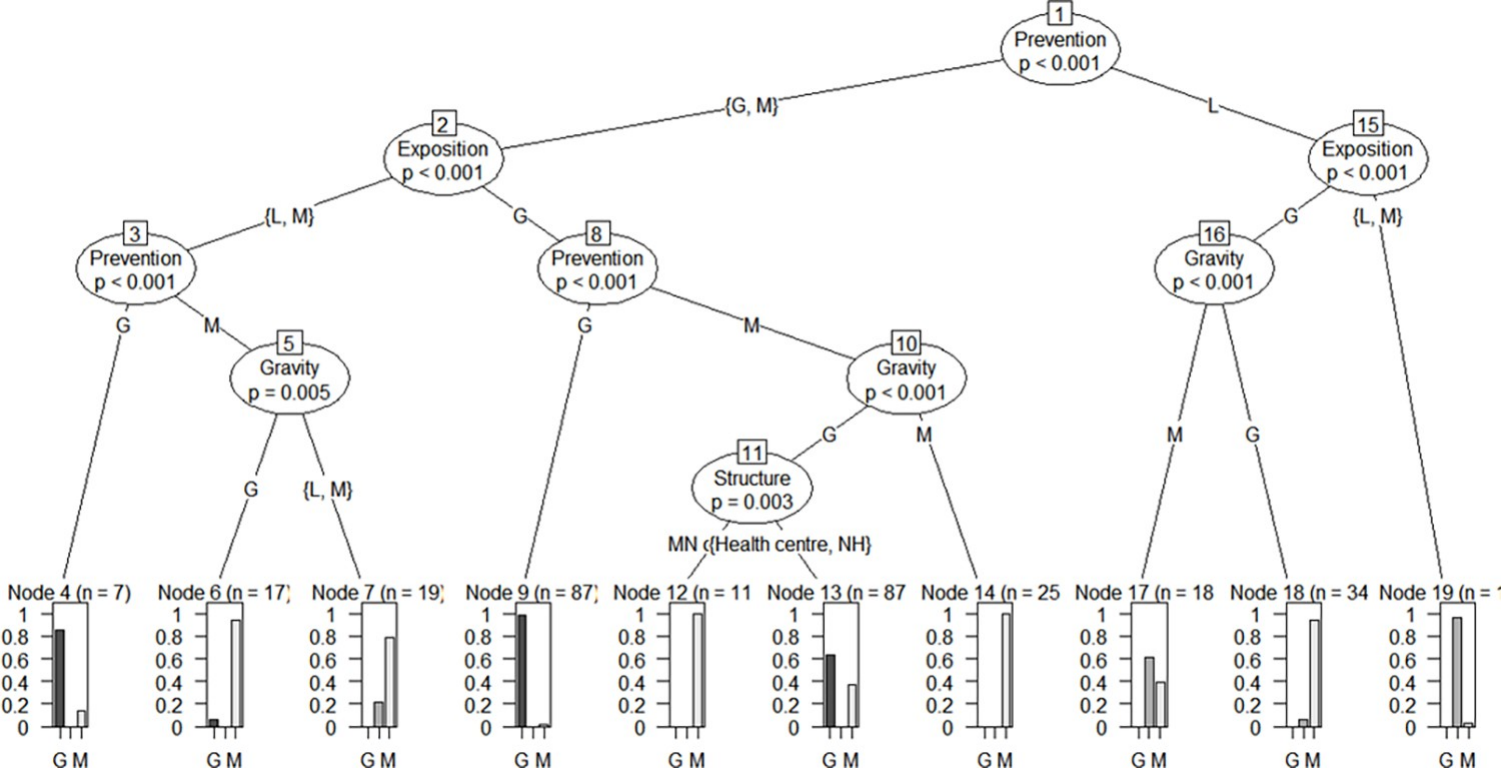
Conditional inference tree on the perceptions of healthcare workers in public health facilities in Conakry Abbreviation: G = Good perception; M = Moderate perception; L = Low perception; MNC = Municipal Hospital and NH = National Hospital.

Respondents’ overall perceptions depended on their awareness regarding the severity, the exposure, the preventive measures for arboviruses, as well as their healthcare structures. The model predicts a good overall perception for respondents with a good perception of measures to prevent arboviruses (p<0.001) (Node 4 and 9). The model shows that HCWs at municipal hospitals had a low overall perception of arboviruses (Node 12). In contrast, those in primary health centers and national hospitals had a good overall perception (Node 13) (Fig 5).

## Discussion

This study described the knowledge, attitudes and perceptions on arboviruses in a population of HCWs in public health facilities in Conakry, Guinea. It was conducted in a non-epidemic context in order to sensitise practitioners to consider arboviruses as a possible diagnosis in symptomatic patients and to better prepare them to the emergence and/or re-emerging of vector-borne viral diseases. To the best of our knowledge, it is the first study in Guinea and even in Africa evaluating the knowledge, attitudes and perceptions on arboviruses among HCWs.

The results showed a low level of knowledge of respondents on arboviruses, particularly the types, vectors and modes of transmission of arboviruses, as well as management and preventive measures. This could partly explain the under-detection of arbovirus cases in health care facilities and the country’s current situation with regard to arbovirus transmission as well as the perceived risk of arboviral diseases outbreaks. Arboviral diseases (dengue, yellow fever, rift valley fever and Crimean Congo haemorrhagic fever) are under surveillance in Guinea. However, the country is often confronted with outbreaks of yellow fever, with deaths occurring in some cases. The last epidemic was reported in November 2022. Other yellow fever epidemics were documented in 2020 [11], 2008 [25] and 2005 [26]. Our results confirm the conclusions of a recent survey of the WHO which assessed the capacities of health systems to prevent, detect and respond to arboviral disease outbreaks in the 47 countries of the WHO African Region. The lack of appropriate training for staff involved in the surveillance and control of arbovirus diseases has been identified by the Department of Neglected Tropical Disease Control of the WHO as one of the recurring gaps that African countries must fill if they are to be adequately prepared against emerging and/or outbreaks of arboviral diseases [27]. The sensitivity of surveillance for vector-borne viral diseases depends on the level of knowledge of HCWs of these diseases, hence the need to further strengthen their capacity to adopt a proactive approach.

The attitudes of HCWs to arbovirus-related diseases differed according to their grade, age and year of experience. Younger, less experienced HCWs had negative attitudes, particularly on the prevention, diagnosis and therapeutic management of arboviruses. This is due to the fact that they are often not confronted with cases of arboviruses in their medical practice, and diagnostic suspicion is not made systematically. In addition, arboviruses share fairly similar clinical signs of acute febrile syndromes with other endemic diseases such as malaria, which is the leading cause of consultation, hospitalisation and death in health facilities [28]. The similarity of clinical symptoms between arbovirus infections and malaria could increase the frequency of errors in diagnosis and treatment of arboviruses, with long-term sequelae such as encephalitis, haemorrhagia, chronic fatigue syndrome and chronic rheumatism [29–31]. The co-circulation of multiple diseases, which often result in clinically indiscernible febrile syndromes, highlights the need for point-of-care diagnostic tests that help practitioners in their differential diagnosis [32].

Despite the good perception of the gravity and risk of exposure, one third of the respondents had a low perception of preventive measures. This result highlights that HCWs are not sufficiently involved in the prevention and surveillance activities of arbovirus diseases. They are important actors in the prevention and control of vector-borne viral diseases, and their awareness of the problems related to these diseases is more than necessary for a better efficacy of this surveillance system. In addition, the prevention and effective management of these diseases depends on how the disease is perceived, which in turn is influenced by the level of knowledge and the availability of information for decision makers [33,34]. The need to inform and sensitise HCWs is the key to improve their perception of arboviruses.

This study provides useful information that may help to guide future intervention and to adapt the surveillance system and strategies for the control and prevention of arboviruses in Guinea. The results also highlight the need for appropriate training for HCWs involved in surveillance in order to improve the early detection of cases in health facilities. In addition, this study used a decision tree-based data mining algorithm to understand the relationship that may exist between the overall knowledge, attitudes and perceptions of HCWs about arboviruses and those in each field, as well as their basic characteristics in order to identify subgroups to target for arbovirus awareness and communication interventions. The choice of decision trees in this study was based on several reasons: first, they are very easy to interpret and provide a clear visual representation of the decision-making process, so it is easier for non-experts to understand the model’s predictions; second, they don’t assume any linearity between patient characteristics and outcome criteria; third, they can provide a measure of the importance of variables, and this enables selection of characteristics and understanding of those that have the greatest influence on the outcome criteria; and finally, they can handle both numerical and categorical data, which is not always the case with other models. However, this study has some limitations. First, it was performed only in a sample of HCWs in public health facilities, and therefore did not include those in private medical centers, even though public-sector staff are the most numerous and heavily involved in monitoring diseases with an epidemic potential. Additionally, this study is also limited by its cross-sectional nature, so that neither the decision trees can establish a causal relationship between the predictor variables and the response variables. Furthermore, this study was not complemented by a qualitative study such as focus groups or interviews, which are important for gathering detailed and additional information from HCWs.

## Conclusion

This study showed the low level of knowledge and a negative attitude about arboviruses on a large sample of HCWs in Conakry, Guinea. Ongoing training programs are more than necessary to enhance the knowledge and skills of HCWs in order to carry out effective surveillance activities, so that cases can be detected in good time to deal with them. In addition, other studies in the different health regions of Guinea using a mixed approach (quantitative and qualitative) will be interesting in order to raise awareness of the HCWs about the risk of arboviruses.

## Supporting information

S1 Questionnaire QUESTIONNAIRE. (PDF)

S1 Data Excel spreadsheet containing, in separate sheets, data relating to respondents’ characteristics, knowledge, attitudes and perceptions, as well as data for Figs 2, 3, 4 and 5. (XLSM)
