# Supplementary material for: Exploratory analysis of the knowledge, attitudes and perceptions of healthcare workers about arboviruses in the context of surveillance in the Republic of Guinea
Source: PLoS Negl Trop Dis. 2023 Dec 4;17(12):e0011814. doi: 10.1371/journal.pntd.0011814 (PMC10721174; doi:10.1371/journal.pntd.0011814)
Supplement: S1 Questionnaire — (PDF) [file pntd.0011814.s001.pdf]

QUESTIONNAIRE

SOCIO-DEMOGRAPHIC CHARACTERISTICS

Q1. Sex	▢ Male<br>▢ Female
Q2. Date of birth (dd/mm/yyyy)	______/______/________/<br>(or note the age): _______ years
Q3. Grade	▢ Specialized clinician<br>▢ General practitioner<br>▢ Medical thesis student
Q4. Years of experience	______/ (years)

KNOWLEDGE

Knowledge of the type of arbovirus, vectors and modes of transmission

Q1. How do you define arboviruses? **(1 point)**
- ☐ Viral diseases transmitted by poultry
- ☐ Viral diseases transmitted by flies
- ☐ Viral diseases transmitted by bushmeat
- ☒ Viral diseases transmitted by arthropods

Q2. Which of these diseases are arboviruses? **(1 point)**

Dengue	☐No	☒Yes	☐Don't know	**(0.25 point)**
Measles	☒No	☐Yes	☐Don't know	**(0.25 point)**
Yellow fever	☐Non	☒Yes	☐Don't know	**(0.25 point)**
Trypanosomiasis	☒Non	☐Yes	☐Don't know	**(0.25 point)**

Q3. Anopheles mosquitoes are the main vectors of arboviruses? **(1 point)**
- ☒No
- ☐Yes
- ☐Don’t know

Q4. Aedes mosquitoes are the main vectors of arboviruses? **(1 point)**
- ☐No
- ☒Yes
- ☐Don’t know

Q5. Are arboviruses zoonoses? **(1 point)**
- ☐No
- ☒Yes
- ☐Don’t know

Q6. Can humans be reservoirs for certain arboviruses? **(1 point)**
- ☐No
- ☒Yes
- ☐Don’t know

Q7. Do climatic factors contribute significantly to the emergence of arboviruses? **(1 point)**
- ☐No
- ☒Yes
- ☐Don’t know

Q8. Are arboviruses chronic diseases? **(1 point)**
- ☒No
- ☐Yes

☐Don't know

Q9. Arboviruses can be transmitted by :

droplets	☒No	☐Yes	☐Don't know **(0.25 point)**		
contact with patients	☒No	☐Yes	☐Don't know **(0.25 point)**		**(1 point)**
blood transfusion	☐No	☒Yes	☐Don't know **(0.25 point)**		
consumption of bushmeat	☒No	☐Yes	☐Don't know **(0.25 point)**		

Q10. Which of these arboviruses are considered to be priority zoonoses in Guinea?

Dengue	☐No	☒Yes	☐Don't know **(0.25 point)**	
Chikungunya	☒No	☐Yes	☐Don't know **(0.25 point)**	**(1 point)**
Rift Valley fever	☐No	☒Yes	☐Don't know **(0.25 point)**	
Yellow fever	☐No	☒Yes	☐Don't know **(0.25 point)**	

Knowledge of the symptoms, diagnosis, treatment and complications of arboviruses

Q11. Influenza-like syndrome is one of the first symptoms of arboviruses? **(1 point)**

☐No
☒Yes
☐Don't know

Q12. Gastrointestinal troubles are the main symptoms of arboviruses? **(1 point)**

☒No
☐Yes
☐Don't know

Q13. Arboviruses are mostly asymptomatic? **(1 point)**

☐No
☒Yes
☐Don't know

Q14. What complications can be caused by arboviruses?

Liver disease	☐No	☒Yes	☐Don't know **(0.25 point)**	
Encephalic syndromes	☐No	☒Yes	☐Don't know **(0.25 point)**	**(1 point)**
Haemorrhagic syndromes	☐No	☒Yes	☐Don't know **(0.25 point)**	
Ocular manifestations	☐No	☒Yes	☐Don't know **(0.25 point)**	

Q15. Can the diagnosis of arboviruses be confirmed by PCR? **(1 point)**

☐No
☒Yes
☐Don't know

Q16. Arboviruses treatment is symptomatic? **(1 point)**

☐No
☒Yes
☐Don't know

Q17. Is there a specific treatment for arboviruses? **(1 point)**

☒No
☐Yes
☐Don't know

Q18. Are antibiotics necessary in the treatment of arboviruses? **(1 point)**

☒No
☐Yes
☐Don't know

Q19. Is chloroquine effective in the treatment of arboviruses? **(1 point)**

☒No
☐Yes
☐Don't know

Knowledge of prevention measures

Q20. Which of these measures will protect against arboviruses? **(1 point)**

Sleeping under mosquito net	☐No	☒Yes	☐Don't know	**(0.25 point)**
Eliminating stagnant water around	☐No	☒Yes	☐Don't know	**(0.25 point)**
Chemoprophylaxis	☒No	☐Yes	☐Don't know	**(0.25 point)**
Wearing long, loose-fitting clothing	☐No	☒Yes	☐Don't know	**(0.25 point)**

Q21. For which arboviruses is there a vaccine? **(1 point)**

Dengue	☐No	☒Yes	☐Don't know	**(0.25 point)**
Chikungunya	☒No	☐Yes	☐Don't know	**(0.25 point)**
Rift Valley fever	☒No	☐Yes	☐Don't know	**(0.25 point)**
Yellow fever	☐No	☒Yes	☐Don't know	**(0.25 point)**

NB: Ticked boxes indicate correct answers.

Each correct answer had a value of "1" and the incorrect or don't know answer had a value of "0". The values were added to obtain a total score from 0 to 21 points which was categorized as follows: good knowledge (80-100%) if the score was between 17 and 21 points, moderate (50-79%) when the score was between 11 and 16 points, poor (<50%) in case of a score less than 11 points.

ATTITUDES

Q1. Arboviruses are a major public health problem? **(1 point)**

☐Disagree
☒Agree
☐Don’t know

Q2. Are you worried that you may be at risk of contracting arboviruses? **(1 point)**

☐Disagree
☒Agree
☐Don’t know

Q3. Can arboviruses be prevented? **(1 point)**

☐Disagree
☒Agree
☐Don’t know

Q4. Can arboviruses be a possible diagnosis in a febrile patient? **(1 point)**

☐Disagree
☒Agree
☐Don’t know

Q5. Can arboviruses be treated using :

a. Antibiotics **(1 point)**

☒Disagree
☐Agree
☐Don’t know

b. Antipyretics/analgesics **(1 point)**

☐Disagree
☒Agree
☐Don’t know

c. Antivirals **(1 point)**

☒Disagree
☐Agree
☐Don’t know

d. Anti-inflammatory **(1 point)**

☐Disagree
☒Agree
☐Don’t know

NB: Ticked boxes indicate correct answers.

Each correct answer was coded as "1" and the incorrect answer or don't know was coded as "0". An overall score above six (80%) was considered as a positive attitude, while a score less than or equal to six (<80%) was considered as a negative attitude.

PERCEPTIONS

Q1. How gravity do you think arboviruses are? *(Please circle the number that corresponds to your level of perception. The number 1 indicates that arboviruses are not at all severe and the number 5 indicates that they are very severe.)*

1	2	3	4	5

Q2. What do you think about mosquito bites? *(Please circle the number that corresponds to your level of perception. The number 1 indicates that mosquito bites are not at all severe and the number 5 indicates that they are very severe.)*

1	2	3	4	5

Q3. What do you think about diseases transmitted by mosquitoes and other arthropods? *(Please circle the number that corresponds to your level of perception. The number 1 indicates that arthropod-borne diseases are not at all severe and the number 5 indicates that they are very severe.)*

1	2	3	4	5

Q4. Do you consider that you are exposed to bites from mosquitoes and other arthropods? *(Please circle the number that corresponds to your level of exposure. The number 1 indicates not at all exposed and the number 5 indicates very exposed)*

1	2	3	4	5

Q5. Are you afraid of mosquito bites and other arthropods? *(Please circle the number that corresponds to your level of exposure. The number 1 indicates not at all and the number 5 indicates completely)*

1	2	3	4	5

Q6. Does the thought of contracting a disease transmitted by mosquitoes and other arthropods have you worried? *(Please circle the number that corresponds to your level of exposure. The number 1 indicates not at all and the number 5 indicates completely)*

1	2	3	4	5

Q7. Do you think you are exposed to diseases transmitted by mosquitoes and other arthropods? *(Please circle the number that corresponds to your level of exposure. The number 1 indicates not at all exposed and the number 5 indicates very exposed)*

**1**	**2**	**3**	**4**	**5**

What do you think of the effectiveness of preventive measures against mosquito bites?

Q8. Skin lotions, sprays and repellent creams *(Please circle the number that corresponds to your level of confidence in the prevention measures. A number 1 indicates that it is not at all effective and a number 5 indicates that it is very effective)*

1	2	3	4	5

Q9. Insecticide bombs in the home *(Please circle the number that corresponds to your level of confidence in the prevention measures. A number 1 indicates that it is not at all effective and a number 5 indicates that it is very effective)*

1	2	3	4	5

Q10. Electrical sockets, insecticide sprays in the home *(Please circle the number that corresponds to your level of confidence in the prevention measures. A number 1 indicates that it is not at all effective and a number 5 indicates that it is very effective)*

1	2	3	4	5

Q11. Sleeping under a mosquito *(Please circle the number that corresponds to your level of confidence in the prevention measures. A number 1 indicates that it is not at all effective and a number 5 indicates that it is very effective.)*

1	2	3	4	5

Q12. Mosquito screens on windows *(Please circle the number that corresponds to your level of confidence in the prevention measures. A number 1 indicates that it is not at all effective and a number 5 indicates that it is very effective.)*

1	2	3	4	5

Q13. Eliminating stagnant water around your home *(Please circle the number that corresponds to your level of confidence in the prevention measures. A number 1 indicates that it is not at all effective and a number 5 indicates that it is very effective.)*

1	2	3	4	5

Q14. Covering water containers and tanks *(Please circle the number that corresponds to your level of confidence in the prevention measures. A number 1 indicates that it is not at all effective and a number 5 indicates that it is very effective.)*

1	2	3	4	5

Q15. Air conditioning *(Please circle the number that corresponds to your level of confidence in the prevention measures. A number 1 indicates that it is not at all effective and a number 5 indicates that it is very effective.)*

1	2	3	4	5

Q16. Wearing long, loose-fitting clothes *(Please circle the number that corresponds to your level of confidence in the prevention measures. A number 1 indicates that it is not at all effective and a number 5 indicates that it is very effective.)*

1	2	3	4	5

NB: Responses were ranked on a scale from 1 to 5. Each participant should circle the number corresponding to their level of perception. The total score varied from 16 to 80 points. The respondents' perceptions were categorized: good perception if the score is between 80 and 100%, moderate if the score is between 50 and 79%, low if the score is less than 50%.

INFORMATION ON ARBOVIRUSES

Q1. Have you received any information about arboviruses?	▢ No<br>▢ Yes

If no, go to question 5.

Q2. If yes, when did you receive this information about arboviruses?	▢ Under a week<br>▢ 1 to 2 week(s)<br>▢ 3 to 4 weeks<br>▢ Several months ago<br>▢ Several years ago
Q3. If yes, which were your main sources of information?<br><br>*Several possible choices*	▢ Colleagues<br>▢ Medical Journal<br>▢ Scientific congress<br>▢ Online media<br>▢ Newspapers<br>▢ Tv<br>▢ Radio<br>▢ Other (please specify): ___________________________
Q4. Do you need information on arboviruses?	▢ No<br>▢ Yes
Q5. If yes, what kind of information on arboviruses would you like to receive?<br><br>*Several possible choices*	▢ Management<br>▢ Prevention measures<br>▢ Mode of transmission<br>▢ Physiopathology<br>▢ Clinical characteristics<br>▢ Policy Directives<br>▢ Epidemiology<br>▢ Other (please specify): __________________________
